# An Insight into the Role of *Trissolcus mitsukurii* as Biological Control Agent of *Halyomorpha halys* in Northeastern Italy

**DOI:** 10.3390/insects11050306

**Published:** 2020-05-14

**Authors:** Davide Scaccini, Martina Falagiarda, Francesco Tortorici, Isabel Martinez-Sañudo, Paola Tirello, Yazmid Reyes-Domínguez, Andreas Gallmetzer, Luciana Tavella, Pietro Zandigiacomo, Carlo Duso, Alberto Pozzebon

**Affiliations:** 1 Department of Agronomy, Food, Natural Resources, Animals and Environment, University of Padova, viale dell’Università 16, 35020 Legnaro, Italy; isabel.martinez@unipd.it (I.M.-S.); paola.tirello@unipd.it (P.T.); carlo.duso@unipd.it (C.D.); 2 Laimburg Research Centre, Laimburg 6, Pfatten (Vadena), IT-39040 Auer (Ora), South Tyrol, Italy; Martina.Falagiarda@laimburg.it (M.F.); Yazmid.Reyes-Dominguez@laimburg.it (Y.R.-D.); andreas.gallmetzer@laimburg.it (A.G.); 3 Dipartimento di Scienze Agrarie, Forestali e Alimentari, University of Torino, largo P. Braccini 2, 10095 Grugliasco, Italy; francesco.trt@gmail.com (F.T.); luciana.tavella@unito.it (L.T.); 4 Department of Agricultural, Food, Environmental and Animal Sciences, University of Udine, via delle Scienze 206, 33100 Udine, Italy; pietro.zandigiacomo@uniud.it

**Keywords:** biological control, egg parasitoid, *Trissolcus mitsukurii*, Pentatomidae, Scelionidae, brown marmorated stink bug

## Abstract

Sustainable strategies such as classical or augmentative biological control are currently being evaluated for the long-term management of the alien invasive pest *Halyomorpha halys* (Stål) (Hemiptera: Pentatomidae). A three-year study carried out in northeastern Italy was performed to investigate the distribution and field performance of the *H. halys* egg parasitoid *Trissolcus mitsukurii* (Ashmead) (Hymenoptera: Scelionidae), in comparison with other parasitoid species. In the study area, adventive populations of *T. mitsukurii* were present since 2016, representing the earliest detection of this species in Europe. *Trissolcus mitsukurii* was the most abundant parasitoid and showed a higher “parasitoid impact” (i.e., number of parasitized eggs over the total number of field-collected eggs) compared to the other species, i.e., *Anastatus bifasciatus* (Geoffroy) (Hymenoptera: Eupelmidae), *Trissolcus* *basalis* (Wollaston) and *Trissolcus* *kozlovi* Rjachovskij (Hymenoptera: Scelionidae). The hyperparasitoid *Acroclisoides sinicus* (Huang and Liao) (Hymenoptera: Pteromalidae) was also recorded. Phylogenetic analysis of *T. mitsukurii* population distinguished two clades, one covering samples from Italy, Japan and China, the other from South Korea. The present study provides promising results for the biological control of a pest that is having a dramatic impact on a wide range of crops worldwide.

## 1. Introduction

The brown marmorated stink bug, *Halyomorpha halys* (Stål) (Hemiptera: Pentatomidae), is a polyphagous pest native to eastern Asia, detected in North America in the 1990s [1], in Europe in the mid-2000s [2], and in South America in 2017 [3]. Feeding on more than 170 host plants, *H. halys* causes serious damage to agricultural crops, ornamental plants, and urban and forest trees [4,5,6,7]. Moreover, *H. halys* can represent a nuisance problem in residential areas during fall and winter, when large numbers of adults invade buildings searching for overwintering sites [1,8,9]. In Italy, *H. halys* was reported in the Emilia Romagna region in 2012, in the Piedmont region in 2013, in the Veneto region and in the Friuli-Venezia Giulia region in 2014, and two years later in the Trentino-Alto Adige region [10,11,12,13,14]. The species is now distributed all over the country [13,15].

In the invaded areas, management strategies to control *H. halys* rely on the use of insecticides and exclusion nets [16,17,18]. However, sustainable strategies such as classical or augmentative biological control may be necessary for a long-term management of the pest. In its native area, *H. halys* populations are exploited by several egg parasitoids belonging to the genera *Anastatus* Motschulsky (Hymenoptera: Eupelmidae), *Ooencyrtus* Ashmead (Hymenoptera: Encyrtidae), *Telenomus* Haliday (Hymenoptera: Scelionidae) and *Trissolcus* Ashmead (Hymenoptera: Scelionidae) [18,19]. Among them, *Trissolcus japonicus* (Ashmead) and *Trissolcus mitsukurii* (Ashmead) were identified as the predominant egg parasitoids of *H. halys* in northern China and Japan, respectively [20,21,22]. Adventive populations of *T. japonicus* were found in North America in 2014 [23], in Switzerland in 2017 [24], and in northwest of Italy in 2018 [25,26], while adventive populations of *T. mitsukurii* were only reported in northern Italy [25,26].

How and when these exotic parasitoids were introduced to Europe is still unclear, and this represents an essential aspect in tracking both *H. halys* and its parasitoid spread [23]. Genetic analyses of European *H. halys* populations revealed multiple invasions from different sources in their native areas [27,28,29], which could potentially be involved in the distribution pathways of egg parasitoids. To test this hypothesis, we analyzed material on *H. halys* egg parasitoid complexes collected in the northeast of Italy, in areas not covered by previous surveys and colonized by a diverse set of *H. halys* haplotypes [28,29]. Assessments of the parasitoid complexes of *H. halys* eggs in northeastern Italy started in 2016, and we compared the impact of the different parasitoid species across three years. Data revealed that *T. mitsukurii* was the dominant species among egg parasitoids of *H. halys*, and we characterized the genetic structure of its population.

## 2. Materials and Methods

### 2.1. Sampling Sites and Laboratory Study

Eight sites in the Veneto region were surveyed every 7–10 days, from June 2016 to October 2018 (Table 1). The selection of sites was based on *H. halys* population levels. Additional observations were performed in other sites located in northeastern Italy: from the beginning of September to October 2018 in the Trentino-Alto Adige region, and in August 2018 in two sites in the Friuli-Venezia Giulia region (Table 1). Field surveys were conducted by searching for *H. halys* eggs on the vegetation, and sampling the canopy between 0.5 to 2 m from the ground, inspecting in particular fruits and leaves; egg masses were hand-collected on the leaves or fruits and transferred to the laboratory. Field-collected egg masses were reared in a climatic chamber at 26 ± 1 °C, 65 ± 5% RH, and 16:8 L:D until parasitoid adult emergence. Emerged parasitoids were stored in 70% ethanol for morphological and molecular identification.

The “exploitation efficiency” (number of parasitized eggs divided by the total number of eggs in the parasitized egg masses [30]) was calculated for each site. On data collected from sites showing a high presence of *H. halys* parasitoids and a steady sampling (i.e., Sites 3, 5, 8 and 9; Table 1), the “discovery efficiency” (number of egg masses from which at least one parasitoid emerged on the total number of sampled egg masses) and the “parasitoid impact” (number of parasitized eggs divided by the total number of field-collected eggs) were calculated [30]. Indexes obtained from data at the latter sites were used to compare performances of the parasitoid species emerged from *H. halys* eggs. These indexes were analyzed using a generalized linear model, assuming a binomially distributed model with a logit link function with the GENMOD procedure of SAS^®^ ver. 9.4 [31]. The effect of parasitoid species was tested using a Wald χ^2^ test (α = 0.05) followed by a Tukey’s test (α = 0.05) on the least-square means. Only species observed in more than one site were included in the analyses.

In the case of the Auer (Ora) site (Site 3), eggs from which no *H. halys* nymphs or parasitoids emerged were further assessed for other causes of mortality [32] under a Leica stereomicroscope (series MZ6) according to Morrison et al. [33], and then ascribed to predation or to other causes of mortality (e.g., deformed and discolored eggs). For eggs where *H. halys* hatching or parasitoid emergence took place in the field before sampling, the category was assessed according to Jones et al. [34], and eggs were classified as parasitized or hatched eggs.

### 2.2. Insect Identification and Molecular Analysis

Ethanol-stored specimens were dried and glued on card points for morphological analyses. A Wild M3 stereomicroscope with magnification up to 200× and a 2700 k spotlight was used for morphological diagnosis. The Palaearctic genera of Scelionidae and *Trissolcus* species were respectively determined using keys from Kozlov and Kononova [35] and Talamas et al. [36]. Moreover, *Trissolcus* specimens were compared with pictures of holotypes and paratypes in Hymenoptera Online (HOL), provided by Talamas et al. [36]. *Anastatus* species were identified using the key by Askew and Nieves-Aldrey [37]. *Acroclisoides* (Hymenoptera: Pteromalidae) specimens were kindly identified by Dr. Mircea-Dan Mitroiu [38]. The parasitoids used for morphological identification were deposited in the Department of Agronomy, Food, Natural Resources, Animals and Environment (DAFNAE), Legnaro, Padua, Italy, in Dipartimento di Scienze Agrarie, Forestali e Alimentari (DISAFA), Grugliasco, Turin, Italy, and in Laimburg Research Center, Auer, Bolzano, Italy.

To confirm the results of the morphological identification for parasitoid species and to study phylogenetic relationship for the most common non-native parasitoid species, molecular analyses were conducted. Total DNAs from three *Trissolcus* adults, singularly analyzed, collected in Trentino-Alto Adige were extracted by homogenizing each sample in 400 µL of CTAB buffer (CTAB 2.5%, Tris pH 8 100 mM, NaCl 1.4 M, EDTA 50 mM pH 8, PVP-40 1%, Proteinase K 10 mg/mL) in a microcentrifuge tube containing a 5-mm tungsten carbide bead (Qiagen, Valencia, CA, USA). Samples were disrupted using a Retsch Mixer Mill MM 400, at 30 Hz for 3 min. After disruption, DNA was extracted using the DNeasy Blood and Tissue kit (Qiagen) following the instructions of the provider. Moreover, DNA of 14 parasitoid samples from Veneto and of three samples from Friuli-Venezia Giulia, singularly analyzed, was extracted according to a previously described salting-out protocol [39]. A partial region of the cytochrome c oxidase subunit I (COI) gene was amplified with primers HCO2198 (5′-GGTCAACAAATCATAAAGATATTGG-3′) and LCO1490 (5′-TAAACTTCAGGGTGACCAAAAAATCA-3′ [40]). PCR amplification of samples analyzed in the Trentino-Alto Adige region was performed in a final volume of 20 µL, containing an equal proportion of Phusion High-Fidelity PCR Master Mix and HF Buffer (New England BioLabs, Ipswich, MA, USA), 200 nM final concentration of each primer and 2 µL of DNA template extraction. Reactions of samples were performed on a Verity 96-Well Thermal Cycler (Applied Biosystems, Foster City, CA, USA) as follows: 30 s at 98 °C, followed by 35 cycles of 50 s denaturation at 98 °C and 90 s for annealing at 52 °C, with 15 s elongation at 72 °C. Amplifications of samples from the Veneto and Friuli-Venezia Giulia regions were performed in 20-μL reactions (1× PCR Go Taq Flexi buffer—Promega, Madison, WI, USA, 2.5 mM MgCl_2_, 0.1 mM dNTPs, 0.5 μM for each primer, 0.5 U of Taq polymerase—Promega, 2 μL DNA template). Thermal cycling conditions were 5 min at 96 °C followed by 4 cycles of 96 °C for 1 min, 47 °C for 1 min, and 72 °C for 1 min, and another 35 cycles of 96 °C for 1 min, 50 °C for 1 min, and 72 °C for 1 min, with a final extension of 72 °C for 5 min.

The produced amplicons were purified through a QIAquick PCR and Gel Cleanup Kit following the instructions of the provider (Qiagen). The purified amplicons were sequenced by LGC Genomics GmbH (Berlin, Germany). PCR products from Veneto and Friuli-Venezia Giulia samples were purified using exonuclease and Antarctic Phosphatase (GE Healthcare, Chicago, IL, USA) and sequenced at the BMR Genomics Service (Padua, Italy).

Sequences were edited and aligned using MEGA X software [41]. To assess the identity of the sequences obtained, the integrated bioinformatics platform Barcode of Life Data System database [42] was used, and a nucleotide Blast was performed using the Basic Local Alignment Search Tool (National Center for Biotechnology Information–NCBI). Similar sequences retrieved from both databases were added to our dataset. Additionally, sequences of the same target gene obtained in previous studies [25,26] were added to our dataset.

To exclude the presence of stop codons in the coding sequences, all COI sequences were translated with Transeq (EMBOSS [43]).

Phylogenetic relationships among sequences of non-native species were estimated with an approximate maximum-likelihood (ML) analysis, using MEGA X software. A GTR + I + G model was applied, with neighbor-joining starting tree and 100 bootstrap replications. A haplotype parsimony network of the final dataset with a probability cut-off at 90% was reconstructed following the TCS method in PopART [44].

## 3. Results

In this study, 251 egg masses and 6527 eggs were collected (Table 2). Egg masses were mostly found on leaves and fruits of crop plants in Veneto, on maple leaves and fruits (93.7%), ailanthus and linden leaves in Trentino-Alto Adige, and on fig and soybean leaves in Friuli-Venezia Giulia (Table 1). In all the three years, *H. halys* egg masses were found in particular from mid-June to the end of September.

Five species of hymenopteran parasitoids emerged from 46 *H. halys* egg masses, and in five cases the egg mass was parasitized by more than one parasitoid species (Table 2). There were no sites with all five parasitoid species together, and, in many cases, only one species was found (“Relative Abundance per Site” in Table 2). These parasitoids emerged mainly between July and September, and some individuals also in June and October.

Based on morphological analyses, *Trissolcus* individuals were identified as *Trissolcus basalis* (Wollaston), *T. kozlovi* Rjachovskij and *T. mitsukurii*. The latter was the most abundant and it emerged from collected egg masses from June (2018) or August (2016) to October, and from May to August in 2017. Both *T. basalis* and *T. kozlovi* were less frequently found parasitizing *H. halys* eggs (Table 2). Six *T. basalis* emerged from egg masses collected at Site 8 on 17 October 2016 and Site 2 on 15–17 August 2018, while two *T. kozlovi* individuals emerged from an egg mass at Site 10 on 22 June 2017 (Table 2). Other Hymenoptera emerged from eggs were morphologically identified as *A. bifasciatus* and the hyperparasitoid *Acroclisoides sinicus* (Huang and Liao) (senior synonym of *A. solus* Grissell and Smith [38]). The first species was found in six sites across Veneto and Trentino-Alto Adige regions, while *A. sinicus* individuals emerged from *H. halys* egg masses in three sites, on 29 August 2017 (nine individuals: Site 8; six individuals: Site 9) and on 27 September 2018 (14 individuals: Site 3; Table 2). These three egg masses were probably primary parasitized by *T. mitsukurii*, as indicated by the presence of round exit holes and the dark ring on the upper border [38].

Parasitoid performances were compared among *A. bifasciatus*, *T. basalis* and *T. mitsukurii* during the three years of observation. No differences were recorded in parasitoid discovery efficiency in the three years (Table 3). The exploitation efficiency among species did not differ in 2016, while, in 2017 and 2018, *T. mitsukurii* showed a higher exploitation efficiency than *A. bifasciatus* (Table 3). Exploitation efficiency varied from 32.3% to 100% for *T. mitsukurii*, and from 7.4% to 46.4% for *A. bifasciatus*, while for the other species it was always below 18.0% (Table 2). Parasitoid impact varied among species in all years, with *T. mitsukurii* showing always the highest value (Table 3). In Site 3, 327 eggs (out of 1520) were found with signs of sucking or chewing by predators. Deformed or discolored eggs were also observed; 939 *H. halys* eggs (61.8%) hatched (Supplementary Table S1).

**Figure 1:**
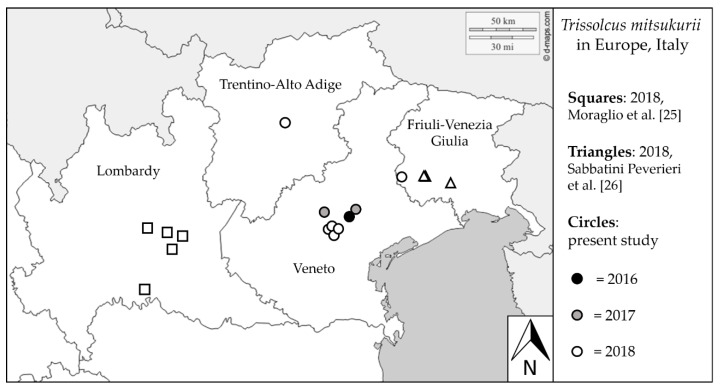
Current distribution of *Trissolcus mitsukurii* in its non-native area in Europe, northern Italy, with first records separated by year. Adapted from www.d-maps.com. Data from Moraglio et al. [25] and Sabbatini Peverieri et al. [26] are included.

*Trissolcus mitsukurii* was recorded in seven sites in Veneto, and in one site in Trentino-Alto Adige and in Friuli-Venezia Giulia. In the Veneto region, the first three records of this parasitoid occurred in 2016 (Site 8) and 2017 (Sites 9 and 11; Figure 1). The presence of *T. mitsukurii* emerging from *H. halys* eggs was recorded in these three sites also in the following year(s). To date, the distribution of *T. mitsukurii* in its non-native area covers many cultivated and natural areas in northern Italy, across at least four regions (Figure 1).

### Molecular Analysis

The results of the molecular analysis were consistent with the morphological identification of *T. basalis*, *T. kozlovi* and *T. mitsukurii*. A total of 20 *Trissolcus* specimens—14 from Veneto, 3 from Trentino-Alto Adige and 3 from Friuli-Venezia Giulia—were successfully amplified and sequenced. A fragment of 534 bp of COI was obtained for all sequences. A BLAST search of sequences from 18 adult insects showed significant alignment with the deposited *T. mitsukurii* sequence AB971831.1, giving an E-value of 0.0, 100% query coverage and 99.32% sequence identity. Similarly, BOLD Systems databases showed a >99% similarity with *T. mitsukurii*. The COI sequence of the *T. kozlovi* specimen in this study had 100% sequence identity to those of Moraglio et al. [25], while *T. mitsukurii* specimens have a perfect match with data in Sabbatini Peverieri et al. [26]. Sequences were deposited in the NCBI database with the GenBank accession numbers MT345599–MT345602.

**Figure 2:**
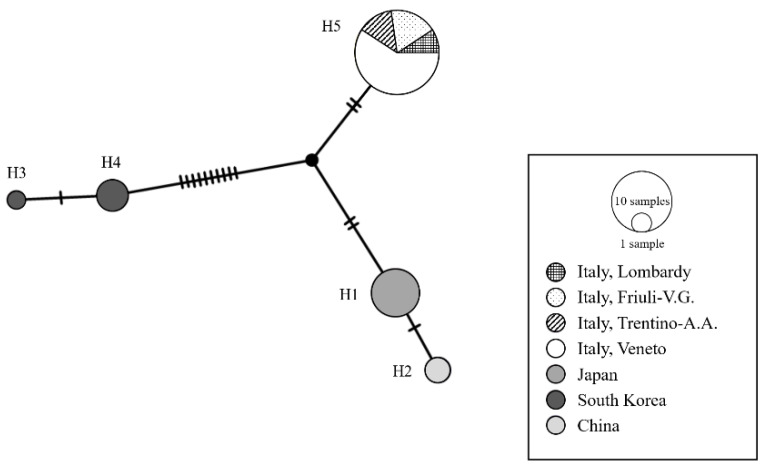
Haplotype network reconstructed with PopART, with the *Trissolcus mitsukurii* cytochrome c oxidase subunit I (COI) sequences obtained in this study and those present in GenBank. Each haplotype is represented by a circle, with the area of the circle proportional to its frequency. Each line represents a single mutation, while crossing lines symbolize missing intermediate or unsampled haplotypes.

**Figure 3:**
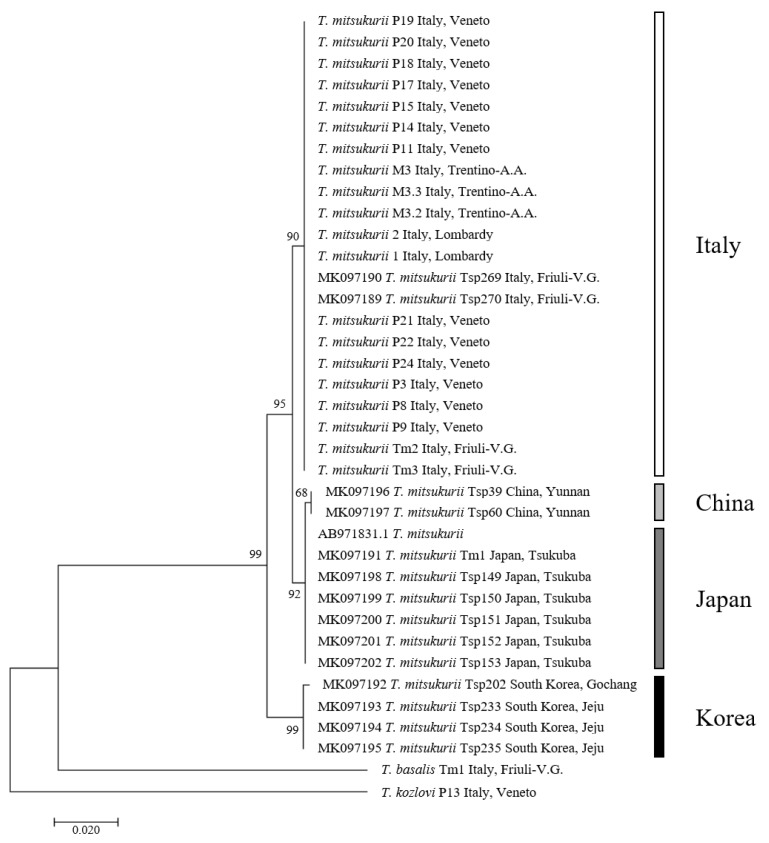
Maximum likelihood (ML) tree based on COI sequences of *Trissolcus mitsukurii*. Numbers on the nodes refer to ML bootstrap values.

The phylogenetic tree, inferred with sequences of this study and sequences retrieved from GenBank database, distinguished between two highly supported clades. One of them grouped the *T. mitsukurii* samples from Italy, Japan and China, while the other clade contained specimens from South Korea (Figure 2 and Figure 3). The TCS Network showed the presence of five haplotypes (Figure 2). The haplotype H5 grouped all sequences of this study and five sequences previously obtained from Italy (MK097188, MK097189 and MK097190 in Sabbatini Peverieri et al. [26], and two sequences from Lombardy—kindly provided by S.T. Moraglio). The other four haplotypes corresponded to sequences from Asian samples. Two of them, H1 and H2, were separated from H5 by four and five mutational steps, respectively, and included samples from Japan and the Yunnan province of China [26,45]. The other two haplotypes, H4 and H3, differed from H5 by several mutational steps (12 and 13 respectively) and grouped sequences from South Korea [26].

## 4. Discussion

In many areas of the invaded range, *H. halys* is causing severe damage to crops. Current pest management strategies are mostly based on chemical control and have often failed in controlling this pest. The implementation of biological control needs to be considered for the management of this invasive pest [5] because the lack of effective natural enemies is one of the main explanations for the high impact of exotic species in invaded territories [46]. In this three-year study, five different parasitic wasp species emerged from *H. halys* eggs: *A. sinicus*, *A*. *bifasciatus*, *T. basalis*, *T. kozlovi* and *T. mitsukurii*, and the last was the most common species found.

*Trissolcus mitsukurii* is an Asian species reported to develop in *H. halys* eggs [20,36]. In this work, for the first time, the performances of *T. mitsukurii* across multiple years were studied outside its native area. It should be noted that information on the performances of this egg parasitoid on *H. halys* in its native range are also limited. *Trissolcus mitsukurii* turned out to be the dominant species among *H. halys* parasitoids, and it showed the highest performances (i.e., exploitation efficiency and parasitoid impact) compared to the other egg parasitoids. In particular, the parasitoid impact of *T. mitsukurii* ranged from 7.7% to 15.1%, while this parameter for the native *A. bifasciatus* was always lower than 4%. Bearing in mind previously published data on other *H. halys* egg parasitoids, the data observed here for *T. mitsukurii* confirm that this exotic parasitoid can have similar or even higher performances as compare to the best native ones, i.e., *A. bifasciatus*, which displayed yearly average parasitism levels from 0.5% to 13.4% [25,47,48]. The performances of *T. mitsukurii* are comparable to those of *T. japonicus*, the other exotic *H. halys* egg parasitoid observed in Europe, which showed a parasitoid impact from 10.5% to 21.5%, measured in one site in northwestern Italy [25]. However, in another study, the parasitoid impact recorded for *T. japonicus* was no more than 2% in three sites [24].

The distribution area of *T. mitsukurii* is wider than previously defined and includes all regions of northern Italy (except Aosta Valley), covering the area across the Friuli-Venezia Giulia, Veneto, Trentino-Alto Adige, Lombardy and Piedmont regions [25,26,49]. All samples of *T. mitsukurii* found in Veneto share the same haplotype of the samples from Trentino-Alto Adige, Lombardy, and Friuli-Venezia Giulia, suggesting a single introduction event of the parasitoid, which possibly followed the invasion pathways of its invasive host. However, multiple introductions of individuals with the same COI haplotype cannot be excluded. The low genetic diversity in *T. mitsukurii* European populations found here and in previous studies [26] may suggest that this species has spread from a limited area in the Veneto region in 2016 to different sites across the whole northern part of Italy, as recorded two years later [25,26]. This information is of importance for tracking the distribution of the parasitoid outside its native range and for a better understating of the evolution of host-parasitoid population dynamics. Samples of *T. mitsukurii* retrieved in Italy are genetically close to the ones from Japan and China, suggesting that this may be their origin, even though some missing haplotypes separate them. Further analyses using other molecular markers and expanding collection sites in their native area will help to improve knowledge of the genetic variability of this parasitoid and delineate its patterns of invasion in Europe. This kind of study has already been performed with *H. halys* [27,28,29,50,51,52], showing high genetic variability in invaded areas and suggesting multiple introduction events.

The occurrence of adventive populations of *T. mitsukurii* in Europe was set one year before the other Asian parasitoids *T. japonicus* [24]. The latter was found emerging from *H. halys* eggs both in Europe and North America [23,24,25,26,53], where this species seems to coexist with *A. bifasciatus*, possibly acting in synergy in the control of *H. halys* [54]. *Trissolcus mitsukurii* and *T. japonicus* are considered key parasitoids of *H. halys* in its native area [18,20,21,22], and their occurrence where *H. halys* is now considered a major pest offers interesting perspectives for its control. However, little is known about *T. mitsukurii*, and the present study provides information on this species in Europe, adding data on the parasitoid impacts in field conditions.

*Trissolcus mitsukurii* represents an example of incidental introduction of an exotic parasitoid of *H. halys*, since no classical biological control program has been performed in Europe. This parasitoid emerged from *H. halys* eggs collected in orchards (organic and conventional), vineyards, and ornamental plants in urban areas, and its potential to track *H. halys* in different habitats is advantageous because *H. halys* is highly polyphagous and is present in these environments [5].

No *T. mitsukurii* emerged from collected egg masses of other stink bugs, such as *Nezara viridula* L. [55], but the association with other pentatomid species cannot be excluded [49]. Although we found *T. mitsukurii* only from eggs of *H. halys*, it is known to attack other pentatomids in Asia, including *Biprorulus bibax* Breddin, *Cuspicona privata* Walker, *Dolycoris baccarum* (L.), *Gonopsis* affinis (Uhler), *Lagynotomus assimulans* (Distant), *Nezara antennata* Scott, *N. viridula*, and *Piezodorus rubrofasciatus* (Fabricius) [56,57,58]. Some of these stink bugs are very common in northern Italy [59,60], where *T. mitsukurii* is likely to attack their eggs. Thus, although *T. japonicus*’s fundamental host range has been already investigated [61,62,63], similar studies are needed for *T. mitsukurii*.

Among the other species that emerged from *H. halys* eggs, *A. bifasciatus* was the second-most frequently found. This is not surprising, since it is a common egg parasitoid of *H. halys* in Europe [25,47,48,64]. *Anastatus bifasciatus* exhibits a wide host range that includes 30 known host species belonging to the orders Hemiptera and Lepidoptera [65,66], and its use has been proposed for augmentative biocontrol against *H. halys* [66,67,68]. However, parasitism rates of *A. bifasciatus* in field conditions are generally low [25,47,48,64,68], and data here reported show a lower impact of this parasitoid compared to *T. mitsukurii*.

In this study, other *Trissolcus* species emerged from *H. halys* eggs. In particular, *T. kozlovi* was obtained from a *H. halys* egg mass in summer 2017. This species is morphologically and genetically similar to *T. japonicus* [25,36], and was first recorded in Italy emerging from eggs of *H. halys* and other stink bugs in 2016 [25]. The acceptance of *H. halys* eggs is here confirmed, but its occurrence in the field was limited, and it showed a very low exploitation efficiency. Furthermore, in two sites, *T*. *basalis* emerged from *H. halys* egg masses. Despite the low exploitation efficiency of the species, the ability of *T*. *basalis* to develop on *H. halys* eggs has to be taken into account because of its wide distribution in Europe and USA [36,69], and it has been reported to parasitize live *H. halys* eggs in the southeastern USA [70].

The hyperparasitoid *A. sinicus* was found to emerge from *H. halys* eggs collected in northeastern Italy, associated with other parasitoid species [25,38]. Pteromalidae belonging to the genus *Acroclisoides* were recorded in different studies acting as hyperparasitoids of Scelionidae [71,72,73]. For instance, Gariepy et al. [71] recorded *A. sinicus* from an egg mass of the pentatomid *Chinavia hilaris* (Say) (= *Acrosternum hilare*), which was found to be primarily parasitized by *Trissolcus* sp. Similarly, *Acroclisoides* sp. emerged from egg masses of *N. viridula*, primarily parasitized by *T. basalis* in Australia [71]. *Acroclisoides sinicus* may also act as hyperparasitoid of *Anastatus* spp. [38]. Little is known about the biology of *A. sinicus* and its influence on the primary parasitoids. Still, its occurrence should be considered in the definition of long-term biocontrol strategies, as showing potential impacts on the primary parasitoid species.

The overall parasitoid impact ranged from 11.2% in 2018 to 19.9% in 2016. However, it should be noted that, in this assessment, we did not take into account damaged and unhatched eggs. This can be the result of predatory activity by natural enemies [32,34,74], but also may derive from the parasitoid-induced host egg abortion or be the result of non-emerged parasitoids [74,75,76,77]. For example, Konopka et al. [77] showed that native parasitoids are not always able to develop inside the eggs of a new host species. In their study, *Trissolcus euschisti* (Ashmead), an indigenous parasitoid of *Podisus maculiventris* (Say) in the US, readily attacks *H. halys* eggs, but it is unable to complete its development. Since we did not assess the level of predation and egg abortion, we can expect that the overall impact of *H. halys* natural enemies is higher than what we estimated in this study. Further research should elucidate these aspects to obtain a full picture of the extent of biological control of *H. halys* by the parasitoid complex considered here. Additionally, the present study was designed to investigate the parasitoid complex of *H. halys* on crops and habitat characterized by a high presence of the pest, but the parasitoid abundance and its performances can vary during the season and differ among habitats and crops; thus, future studies should investigate the effects of these factors.

## 5. Conclusions

Ecological interactions between *H. halys* and its parasitoids in the field need to be studied for the selection of effective species as biocontrol agents. The present study, performed on the egg parasitoid complex of *H. halys* in northern Italy, reported high parasitoid impacts by *T. mitsukurii*, and provides promising results for the control of a pest that is having a dramatic impact on a wide range of crops. Data here presented, coupled with those of previous research, provide information on the expanding distribution range of *T. mitsukurii*. However, its distribution appears spottier than evenly distributed, since there are sites in the Veneto region where only a limited number of native parasitoids are recorded. Biological control with egg parasitoids is likely to have an impact on *H. halys* populations, but further studies are needed to assess the host range of *T. mitsukurii* in Europe and which factors influence the distribution of this exotic species.

## Figures and Tables

**Table 1:** Collection sites of *Halyomorpha halys* egg masses in Italy, listed in alphabetic order by region and province.

Site Number	Region	Province	Coordinates, Altitude (m a.s.l.)	Survey Period	Host Plants	Habitat Description
1	Friuli-Venezia Giulia	Pordenone	45.975556 N 12.451667 E, 98 m	August (2018)	*Ficus carica* (on leaves)	Organic orchard (*Ficus carica*)
2	Friuli-Venezia Giulia	Udine	46.032500 N 13.226944 E, 92 m	August (2018)	*Glycine max* (on leaves)	Experimental farm with soybean, other raw crops, vineyards and orchards
3	Trentino-Alto Adige	Bolzano	46.362028 N 11.298500 E, 224 m	September and October (2018)	*Acer* spp. (on leaves and fruits), *Ailanthus altissima* (on leaves) and linden (on leaves)	Urban area (parking zone) with maple trees (*Acer platanoides*, *Acer negundo* and *Acer pseudoplatanus*), ailanthus (*Ailanthus altissima*) and linden (*Tilia platyphyllos*)
4	Veneto	Padua	45.621319 N 11.719279 E, 43 m	June to November (2017–2018)	*Actinidia deliciosa* (on leaves and fruits)	Small organic orchard (*Actinidia deliciosa*), near urban area
5	Veneto	Padua	45.632120 N 11.799309 E, 40 m	June to November (2017–2018)	*Actinidia deliciosa* (on leaves and fruits)	Small organic orchard (*Actinidia deliciosa*), near urban area
6	Veneto	Padua	45.646779 N 11.740218 E, 50 m	August to October (2016), June to November (2017)	*Actinidia deliciosa* (on leaves and fruits)	Small organic orchard (*Actinidia deliciosa*), near urban area
7	Veneto	Padua	45.580714 N 11.787064 E, 27 m	August to October (2016), June to November (2017–2018)	*Actinidia deliciosa* (on leaves and fruits)	Organic orchard (*Actinidia deliciosa*), near urban area
8	Veneto	Treviso	45.715497 N 11.939604 E, 61 m	August to October (2016), June to November (2017–2018)	*Actinidia deliciosa*, *Prunus* spp., *Vitis vinifera* (on leaves)	Conventional farm with orchard (*Actinidia deliciosa*, *Malus domestica*, *Prunus avium*, *Prunus persica*, *Prunus* spp.), small vineyard and vegetables, with hedges
9	Veneto	Treviso	45.760649 N 12.007810 E, 94 m	June to November (2017–2018)	*Vitis vinifera* (on leaves)	Conventional farm with vineyard and orchard (*Actinidia deliciosa*, *Malus domestica, Prunus persica*)
10	Veneto	Treviso	45.795083 N 12.440934 E, 17 m	June to October (2017)	*Vitis vinifera* (on leaves)	Conventional farm with vineyard
11	Veneto	Vicenza	45.75157 N 11.68244 E, 100 m	August to October (2016–2017)	*Olea europaea* and *Vitis vinifera* (on leaves)	Conventional farm with *Olea europaea* and *Vitis vinifera*

**Table 2:** Identity and abundance of *Halyomorpha halys* parasitoids and hyperparasitoids emerged from collected egg masses per site.

Site Number	Total *n* Egg Masses	Total *n* Eggs	Mean (± SE) of Eggs/Egg Mass	*n* of Parasitized Egg Masses	Parasitoid Species	*n* of Parasitoid Specimens	Relative Abundance per Site (%) ^a^	Exploitation Efficiency (%, Mean ± SE) per Egg Mass (*n* of Egg Masses) ^b^	*n* of Egg Masses with More Than a Parasitoid Species
1	1	28	28	1	*Trissolcus mitsukurii*	24	100	85.71 (1)	
2	2	55	27.5 (± 0.7)	1	*Trissolcus basalis*	1	100	3.57 (1)	
3 ^c^	63	1520	24.1 (± 0.2)	22	*Acroclisoides sinicus*	14	10.4	100 (1)	2 (*A. bifasciatus* and *T. mitsukurii*)
					*Anastatus bifasciatus*	66	49.3	31.1 ± 10.4 (11)	
					*Trissolcus mitsukurii*	54	40.3	43.5 ± 16.4 (5)	
4	10	271	27.1 (± 0.1)	1	*Trissolcus mitsukurii*	26	100	100 (1)	
5	9	241	26.8 (± 0.1)	2	*Anastatus bifasciatus*	8	25.8	28.6 (1)	
					*Trissolcus mitsukurii*	23	74.2	85.2 (1)	
6	22	583	26.5 (± 0.1)	3	*Anastatus bifasciatus*	18	40.0	27.1 ± 8.6 (2)	1 (*A. bifasciatus* and *T. mitsukurii*)
					*Trissolcus mitsukurii*	27	60.0	32.3 ± 13.8 (2)	
7	32	838	26.2 (± 0.1)	2	*Anastatus bifasciatus*	13	59.1	46.4 (1)	
					*Trissolcus mitsukurii*	9	40.9	34.6 (1)	
8	82	2173	26.5 (± 0.2)	8	*Acroclisoides sinicus*	9	5.6	23.1 (1)	1 (*A. bifasciatus* and *T. mitsukurii*)
					*Anastatus bifasciatus*	6	3.7	10.7 (1)	
					*Trissolcus basalis*	5	3.1	17.9 (1)	
					*Trissolcus mitsukurii*	142	87.7	53.7 ± 12.0 (6)	
9	19	513	27.0 (± 0.1)	3	*Acroclisoides sinicus*	6	8.5	22.2 (1)	1 (*A. sinicus* and *T. mitsukurii*)
					*Trissolcus mitsukurii*	65	91.5	80.6 ± 7.8 (3)	
10	5	137	27.4 (± 0.2)	1	*Trissolcus kozlovi*	2	100	7.1 (1)	
11	6	168	28.0 (± 0.1)	2	*Anastatus bifasciatus*	2	7.7	7.4 (1)	
					*Trissolcus mitsukurii*	24	92.3	82.8 (1)	

^a^ As number of parasitoids for each species over the number of all parasitoids found in the site; ^b^ As number of parasitized eggs by a species over the total number of eggs of the parasitized egg mass; ^c^ In this site, some parasitoids emerged from egg masses were not identified (emerged before the collection of the egg mass).

**Table 3:** Average value of discovery efficiency, exploitation efficiency and parasitoid impact on *Halyomorpha halys* egg masses, by parasitoid species observed in the three years.

Index ^a^	Species	Year														
		2016					2017					2018				
		Index		χ^2^	df	*p*-Value	Index		χ^2^	df	*p*-Value	Index		χ^2^	df	*p*-Value
Discovery efficiency (%)	*Trissolcus basalis*	7.7	a	4.02	2	0.1343	n.a.	-	0.79	1	0.3730	n.a.	-	1.17	1	0.2798
	*Trissolcus mitsukurii*	30.8	a				17.3	a				13.2	a			
	*Anastatus bifasciatus*	7.4	a				4.8	a				16.1	a			
Exploitation efficiency (%)	*Trissolcus basalis*	17.9	a	5.47	2	0.0648	n.a.	-	29.47	1	<0.0001	n.a.	-	8.68	1	0.0032
	*Trissolcus mitsukurii*	46.6	a				70.2	a				52.7	a			
	*Anastatus bifasciatus*	38.0	a				10.7	b				37.9	b			
Parasitoid impact (%)	*Trissolcus basalis*	1.5	b	61.68	2	<0.0001	n.a.	-	60.38	1	<0.0001	n.a.	-	11.31	1	0.0008
	*Trissolcus mitsukurii*	15.1	a				9.6	a				7.7	a			
	*Anastatus bifasciatus*	3.3	b				0.1	b				3.5	b			

^a^ For each index, values followed by the same letter are not significantly different to the Tukey’s test on the least-square means (α = 0.05).

## Supplementary Materials

The following are available online at https://www.mdpi.com/2075-4450/11/5/306/s1, Table S1: Overview of fates and parasitism of *Halyomorpha halys* egg masses collected in the study site of Auer (Ora), Trentino-Alto Adige (Site 3) in September and October 2018.
