# Supplementary material for: An Insight into the Role of Trissolcus mitsukurii as Biological Control Agent of Halyomorpha halys in Northeastern Italy
Source: Insects. 2020 May 14;11(5):306. doi: 10.3390/insects11050306 (PMC7290990; doi:10.3390/insects11050306)
Supplement: Supplementary file 1 [file insects-11-00306-s001.pdf]

Table S1. Overview on fates and parasitism of *Halyomorpha halys* egg masses collected in the study site of Auer (Ora), Trentino-Alto Adige (Site 3) in September and October 2018.

Sampling Period (2018)	*n* of *H. halys* Eggs Collected	Mean *n* of Eggs/Egg Mass	*n* Eggs Hatched	*n* eggs Predated, Deformed or Discolored [a]	*n* Eggs Parasitized	Parasitoid Impact (%)
10–16 September	664	24.6	515	96	53	8.0
17–23 September	25	25.0	0	21	4	16.0
24–30 September	407	22.6	188	93	126	31.0
1–7 October	210	26.3	131	57	22	10.5
8–14 October	171	24.4	75	47	49	28.7
22–28 October	43	21.5	30	13	0	0
Total	1520	24.1	939	327	254	16.7

[a] Eggs with signs of sucking or chewing predators, deformed or discolored eggs, where the cause of mortality could not be assigned.
